# Fatores sociodemográficos e comportamentais da obesidade: um estudo longitudinal

**DOI:** 10.1590/0102-311XPT103623

**Published:** 2024-07-29

**Authors:** Bianca Mitie Onita, Jaqueline Lopes Pereira, Grégore Iven Mielke, João Paulo dos Anjos Souza Barbosa, Regina Mara Fisberg, Alex Antonio Florindo

**Affiliations:** 1 Faculdade de Saúde Pública, Universidade de São Paulo, São Paulo, Brasil.; 2 School of Public Health, The University of Queensland, Brisbane, Australia.; 3 Grupo de Estudos e Pesquisas Epidemiológicas em Atividade Física e Saúde, Universidade de São Paulo, São Paulo, Brasil.; 4 Escola de Artes Ciências e Humanidades, Universidade de São Paulo, São Paulo, Brasil.

**Keywords:** Obesidade, Estudos Longitudinais, Epidemiologia Nutricional, Obesity, Longitudinal Studies, Nutritional Epidemiology, Obesidad, Estudios Longitudinales, Epidemiología Nutricional

## Abstract

O objetivo foi investigar a associação entre fatores sociodemográficos e comportamentais e a ocorrência da obesidade entre 2014 e 2021 em adultos da cidade de São Paulo, Brasil. Realizou-se estudo prospectivo com 1.241 adultos paulistanos, com 18 anos ou mais, participantes da coorte *Inquérito de Saúde de São Paulo* (ISA) - Atividade Física e Ambiente. O desfecho foi obesidade (sim/não), classificada por meio do índice de massa corporal e com pontos de corte específicos para cada faixa etária. As variáveis de exposição foram: sexo, idade, escolaridade, cor da pele, estado marital, coordenadoria regional de saúde, atividade física nos quatro domínios e comportamentos sedentários. Foram utilizados modelos de regressão logística multinível para a análise longitudinal. Houve aumento significativo de 27,7% na prevalência de obesidade (de 22,6% para 28,9%). Pessoas que praticavam ao menos 150 minutos semanais de atividade física no lazer (OR = 0,44; IC95%: 0,26; 0,76), entre 10 e 150 minutos semanais de atividade física de deslocamento (OR = 0,49; IC95: 0,30; 0,80) e sem companheiro(a) (OR = 0,47; IC95%: 0,28; 0,78) tiveram menos chances de ter obesidade. Pessoas entre 40 e 59 anos (OR = 5,00; IC95%: 2,02; 12,38) e de cor de pele preta (OR = 4,70; IC95%: 1,85; 11,95) apresentaram maiores chances de ter obesidade. O estudo identificou um aumento na prevalência de obesidade durante o período, com aumento nas chances para pessoas de meia idade e cor da pele preta, e diminuição nas chances para pessoas que vivem sem companheiro(a) e para praticantes de atividades físicas no lazer e como forma de deslocamento. Esses resultados podem contribuir para dar suporte a programas e políticas para o controle da obesidade.

## Abstract (en)

This study aimed to investigate the association between sociodemographic and behavioral factors and obesity from 2014 to 2021 in adults in São Paulo city, Brazil. A prospective study was carried out with 1,241 adults aged 18 years or above who lived in São Paulo and participated in the *São Paulo Health Survey* cohort (ISA) - Physical Activity and Environment. The outcome was obesity (yes/no), classified by body mass index and specific cut-off points for each age group. The exposure variables included sex, age, education, skin color, marital status, regional health coordination, physical activity in the four domains, and sedentary behaviors. Multilevel logistic regression models were used for longitudinal analysis. This study found a significant increase (27.7%) in the prevalence of obesity (from 22.6% to 28.9%). People who practiced at least 150 minutes of leisure-time physical activity per week (OR = 0.44; 95%CI: 0.26; 0.76), between 10 and 150 minutes per week of commuting physical activity (OR = 0.49; 95%CI: 0.30; 0.80), and had no partner (OR = 0.47; 95%CI: 0.28; 0.78) were less likely to be obese. People aged from 40 to 59 years (OR = 5.00; 95%CI: 2.02; 12.38) and who were black (OR = 4.70; 95%CI: 1.85; 11.95) were more likely to be obese. This study found an increase in the prevalence of obesity during the studied period, with increased odds for middle-aged and black people and decreased odds for those without a partner and those who practice physical activities in their leisure and as a form of commuting. These results can contribute to support programs and policies to control obesity.

## Resumen

El objetivo fue investigar la asociación entre factores sociodemográficos y conductuales y la presencia de la obesidad entre 2014 y 2021 en adultos de la ciudad de São Paulo, Brasil. Se realizó un estudio prospectivo con 1.241 adultos paulistanos, con 18 años o más, participantes de la cohorte *Encuesta de Salud de São Paulo* (ISA) - Actividad Física y Ambiente. El resultado fue obesidad (sí/no), clasificada a través del índice de masa corporal y con puntos de corte específicos para cada grupo de edad. Las variables de exposición fueron: sexo, edad, escolaridad, color de la piel, estado civil, coordinación regional de salud, actividad física en los cuatro dominios y comportamientos sedentarios. Para los análisis longitudinales se utilizaron modelos de regresión logística multinivel. Hubo un aumento significativo del 27,7% en la prevalencia de la obesidad (del 22,6% al 28,9%). Las personas que practicaron al menos 150 minutos semanales de actividad física por ocio (OR = 0,44; IC95%: 0,26; 0,76), entre 10 y 150 minutos semanales de actividad física de desplazamiento (OR = 0,49; IC95%: 0,30; 0,80) y sin compañero(a) (OR = 0,47; IC95%: 0,28; 0,78) tuvieron menos probabilidades de tener obesidad. Las personas entre 40 y 59 años (OR = 5,00; IC95%: 2,02; 12,38) y de color de piel negra (OR = 4,70; IC95%: 1,85; 11,95) presentaron mayores probabilidades de tener obesidad. El estudio identificó un aumento en la prevalencia de la obesidad durante ese período, con un aumento en las posibilidades para las personas de mediana edad y color de la piel negra, y una disminución en las posibilidades para las personas que viven sin pareja y para las que realizan actividades físicas por ocio y como forma de desplazamiento. Estos resultados pueden contribuir a apoyar los programas y políticas para el control de la obesidad.

## Introdução

A obesidade é uma das doenças crônicas não transmissíveis com maior prevalência no mundo, apresentando etiologia multifatorial e complexa ^1^. Além de doença, a obesidade também é fator de risco para diversas outras doenças como diabetes mellitus, doenças cardiovasculares, hipertensão arterial, cânceres, problemas respiratórios e osteoartrite ^2^, contribuindo na mortalidade precoce, nos anos de vida perdidos por incapacidade e sobrecarga dos sistemas de saúde ^2^.

O reflexo dessa complexidade é observado no constante aumento da sua prevalência e no reconhecimento de ser um problema de saúde pública mundial. Contudo, esse aumento não é homogêneo, ocorrendo de forma mais rápida em países de baixa renda e mais lenta em países de alta renda ^3^. O Brasil, que é classificado como país de renda média-alta de acordo com o Banco Mundial ^4^, registrou aumento de 90% na prevalência de obesidade em adultos no período de 13 anos, passando de 11,8%, em 2006, para 22,4% em 2021 ^5^. A cidade de São Paulo, maior do país em população e que está entre as cidades mais populosas do mundo, duplicou a sua prevalência de obesidade entre os adultos, passando de 11,2%, em 2006, para 22,5% em 2021 ^5^.

Existe um conjunto de fatores associados à obesidade. Entre eles, destacam-se comportamentos como hábitos alimentares não saudáveis, baixos níveis de atividade física e comportamento sedentário ^2^. Entre os fatores sociais e demográficos, evidências demonstram que há uma relação com a idade, expondo que, com o envelhecer, há um aumento da prevalência de obesidade até determinadas etapas da fase da terceira idade ^6^. Ademais, estudos de tendência global demonstram que mulheres apresentam maior prevalência de obesidade quando comparadas aos homens ^6^. Porém, essa diferença não é homogênea nos países ^7^, assim como a relação da obesidade com o nível educacional e renda, variando de acordo com os níveis socioeconômicos dos países ^7^.

Mundialmente, são observados esforços visando a estagnação do aumento da obesidade. Porém, de acordo com o *Relatório Global de Nutrição* de 2021 ^8^, nenhum país está na direção do êxito. Isso expõe a necessidade de evidências atualizadas e robustas do contexto de cada país para direcionar melhor os planos e as ações, principalmente evidências oriundas de estudos longitudinais para orientar políticas públicas. Portanto, é importante destacar certas lacunas, denotando a alta contribuição de estudos de países de alta renda quando comparados aos de baixa e média renda ^9^, sendo em sua maioria com desenho transversal, incluindo estudos brasileiros, o que limita o esclarecimento dos fatores associados ao acometimento da obesidade. Além disso, grande parte dos estudos que avaliam a relação entre obesidade e atividades físicas expõem a relação entre as atividades físicas no lazer ou total de forma transversal, sem se aprofundar em outros domínios que são importantes, como o do deslocamento ou do transporte ^10^.

Visando obter evidências mais robustas para compreender melhor os fatores associados à obesidade na cidade mais populosa do Brasil, os objetivos deste estudo foram: (i) avaliar o acometimento da obesidade em adultos paulistanos durante o período de 2014 a 2021; e (ii) identificar os fatores sociais, demográficos e comportamentais associados longitudinalmente à obesidade, incluindo as práticas de atividades físicas nos diferentes domínios e o comportamento sedentário.

## Métodos

### Delineamento, local e população do estudo

Estudo longitudinal com dados do *Inquérito de Saúde de São Paulo* (ISA) - Atividade Física e Ambiente, composto por residentes da área urbana do Município de São Paulo, Brasil, com uma população de quase 11,5 milhões de habitantes em todo o seu território municipal em 2022 ^11^. O estudo ISA - Atividade Física e Ambiente foi aprovado pelo Comitê de Ética da Escola de Artes, Ciências e Humanidades da Universidade de São Paulo (protocolo nº 10396919.0.0000.5390) e pelo Comitê de Ética da Secretaria Municipal de Saúde de São Paulo (protocolo nº 10396919.0.3001.0086). Mais detalhes podem ser obtidos em publicação anterior ^12^.

### Processo de amostragem e amostra

A amostragem da linha de base foi probabilística por conglomerados e estratificada em dois estágios. Nessa coleta (2014-2015), foram entrevistadas 4.042 pessoas, incluindo homens e mulheres, sendo uma amostra representativa da população com 12 anos ou mais residentes em São Paulo ^13^. Dessas, 3.410 tinham 18 anos ou mais de idade. Para a segunda onda (2020-2021), buscou-se entrevistar todas as pessoas com 18 anos ou mais no momento da coleta, sendo elegíveis adultos que residiam no Município de São Paulo e excluídas mulheres gestantes, pessoas que se mudaram do município, pessoas incapazes de responder o questionário sozinhas devido à alguma alteração cognitiva, pessoas que sofreram acidentes ou cirurgias que os deixaram incapazes de exercer suas atividades habituais/rotineiras e pessoas que utilizam cadeira de rodas de forma permanente. Dos 4.042 indivíduos da linha de base, a segunda onda contou com 1.434 indivíduos adultos, cerca de 35,5% da linha de base.

**Figura 1:**
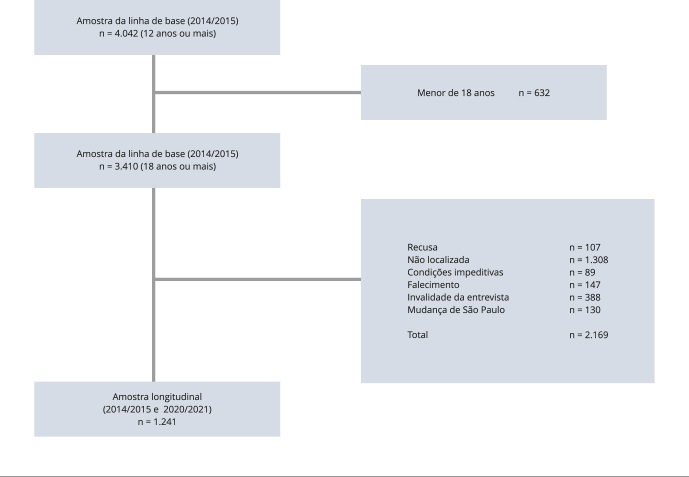
Fluxograma da amostra de adultos do estudo longitudinal. ISA - Atividade Física e Ambiente.

Para este estudo, foram considerados somente indivíduos com 18 anos ou mais já na linha de base. Dos 3.410 indivíduos, 107 se recusaram a participar, 1.308 não foram localizados em tentativas de contato, 477 apresentaram invalidade da entrevista ou alguma condição impeditiva, 147 faleceram, e 130 se mudaram do município. Portanto, a amostra final analisada foi de 1.241 indivíduos adultos (Figura 1). Nas análises comparativas da amostra selecionada (n = 1.241) com as perdas (n = 2.169), foram identificadas diferenças significativas nas variáveis de escolaridade (p = 0,002), sexo (p = 0,021), na idade (p < 0,001) e na prevalência de obesidade (p = 0,006). A amostra das perdas continha mais indivíduos de menor escolaridade, mais homens, mais jovens e indivíduos sem obesidade.

### Coleta de dados

A coleta de dados da linha de base foi realizada por entrevistadores treinados, por meio de entrevistas domiciliares com aplicação de questionário contendo 26 blocos temáticos. O treinamento e a capacitação dos entrevistadores da linha de base incluiu aulas sobre os temas abordados com prova teórica e aulas práticas de conduta e aplicação do questionário ^14^. A coleta da segunda onda do estudo sofreu alterações devido ao acometimento da pandemia de COVID-19 e ocorreu com aplicação de questionário via telefone, também com entrevistadores treinados, seguindo o protocolo já consolidado e adotado pelo Sistema de Vigilância de Fatores de Risco e Proteção para Doenças Crônicas por Inquérito Telefônico (Vigitel) ^15^. O treinamento e a capacitação dos entrevistadores da segunda onda ocorreu de forma remota, com exposição de aula teórica sobre os temas abordados e orientações de conduta e aplicação do questionário no sistema.

### Variável desfecho

O desfecho foi obesidade (sim, não) definida por meio do cálculo do índice de massa corporal (IMC) com as informações de peso e altura autorreferidos, método validado anteriormente, calibrados de acordo com o estado nutricional e o sexo do indivíduo para melhor acurácia dessas informações ^16^. Para a classificação de obesidade, foram utilizados pontos de cortes de acordo com a faixa etária: (i) IMC para idade e sexo, em indivíduos com menos de 20 anos, com ponto de corte +2 ou mais desvios padrão do IMC ^17^; (ii) IMC para adultos e idosos, em indivíduos com 20 anos ou mais, com ponto de corte IMC ≥ 30kg/m^2^ para ambos ^18^ ^,^ ^19^.

### Variáveis de exposição

(1) Atividade física semanal em cada um dos quatro domínios (trabalho, deslocamento, doméstica e lazer), coletadas pelo *Questionário Internacional de Atividade Física* - versão longa (IPAQ longo), questionário validado tanto para coleta presencial quanto telefônica ^20^. Cada domínio foi categorizado em: práticas de menos de 10 minutos semanais; práticas de pelo menos 10 minutos e menos de 150 minutos semanais; e práticas de pelo menos 150 minutos semanais ^21^. As atividades físicas vigorosas de cada domínio foram multiplicadas por dois para computar os minutos semanais de atividades físicas.

(2) Tempo diário de comportamento sedentário geral e assistindo televisão, coletados por questionário validado ^22^, e utilizando o tempo diário ponderado dos comportamentos (considerando cinco dias de semana e dois dias de final de semana). Elas foram categorizadas em: permanece menos de três horas diárias; e permanece pelo menos três horas diárias realizando cada comportamento.

(3) Características sociais e demográficas: provenientes do bloco de características socioeconômicas dos questionários aplicados. As variáveis utilizadas foram: (i) sexo, coletada como feminino ou masculino; (ii) idade, calculada a partir da informação da data de nascimento do indivíduo e categorizada em 18 a 29 anos, 30 a 39 anos, 40 a 59 anos, 60 anos ou mais; (iii) escolaridade, coletada pela pergunta “Até que ano da escola o(a) Sr.(a) completou?” e categorizada em até o 5º ano do Ensino Fundamental, 6º ano do Fundamental a 2ª série do Ensino Médio, Ensino Médio completo, Ensino Superior ou Pós-graduação incompleto/completo; (iv) cor da pele, coletada originalmente com as opções branca, preta, amarela, parda, indígena, outra e categorizada em branca, preta, amarela, parda, outra; (v) estado marital, coletada originalmente com as opções casado no civil ou religioso, vive em união conjugal estável ou vive junto, solteiro, separado, desquitado ou divorciado, viúvo e categorizada como vive com companheiro(a) ou vive sem companheiro(a).

Também foi utilizada a variável de Coordenadoria Regional de Saúde da residência do indivíduo, coletada por meio do georreferenciamento do endereço de sua moradia e categorizada na sua classificação vigente: centro, leste, norte, oeste, sudeste e sul.

### Análises de dados

Para as análises descritivas, foram utilizadas medidas de frequência absoluta e relativa em cada período. Para verificar as diferenças entre as características na linha de base e na segunda onda, foi realizada análise de McNemar (para variáveis dicotômicas e para a prevalência de obesidade) e teste de Wilcoxon pareado (para variáveis categóricas). Em seguida, foram realizadas análises bivariadas, por meio do qui-quadrado de Pearson, para verificar as variáveis associadas à obesidade em cada tempo. Também foram analisados os intervalos de 95% de confiança (IC95%) da prevalência de obesidade em cada categoria das variáveis sociodemográficas e comportamentais, para identificar mudanças no seu acometimento entre a linha de base e a segunda onda. A mudança foi considerada significativa quando os valores dos intervalos não se sobrepunham.

Nas análises longitudinais ajustadas, foi utilizada a análise de regressão logística multinível. Para isso, foi realizado o pareamento dos dados e estruturado o modelo utilizando dois níveis: (i) os indivíduos (n = 1.241), pois ele leva em consideração a dependência das observações intrassujeitos; e (ii) o número total de observações (n = 2.482), pois cada indivíduo apresenta duas medidas ou observações. Em seguida, foram inseridas nos modelos multiníveis longitudinais todas as variáveis que apresentaram valor de p < 0,20 na análise bivariada em cada tempo e variáveis conceitualmente importantes ^23^. A variável tempo (linha de base e segunda onda) foi inserida como ajuste. Foi utilizado o teste de Wald para avaliar as variáveis associadas ao desfecho nos modelos.

Para verificar a significância das variáveis nas análises, adotou-se valores de p < 0,05 e os valores de *odds ratio* (OR) com seus respectivos IC95%. Todas as análises foram conduzidas no software Stata, versão 16.1 (https://www.stata.com).

## Resultados

A amostra, de 1.241 adultos paulistanos, foi composta por maioria do sexo feminino (60%) e de cor de pele branca (52,3%). Em ambos os momentos, houve maior proporção de indivíduos com 40 a 59 anos ou 60 anos ou mais (65,4% na linha de base e 76,1% na segunda onda), com pelo menos o Ensino Médio completo ou Superior completo/incompleto (51,3% na linha de base e 57,8% na segunda onda), que vivem com companheiro(a) (58,7% na linha de base e 57,9% na segunda onda) e residem na região sul do município (24,3% na linha de base e 24,8% na segunda onda) (Tabela 1). Ao comparar a linha de base com a segunda onda, houve aumento de indivíduos com 60 anos ou mais e com o Ensino Médio completo e Superior completo ou incompleto. Quanto às características comportamentais, tanto na linha de base quanto na segunda onda foram observadas maior proporção de indivíduos que praticavam menos de 10 minutos por semana de atividades físicas no lazer (68,3% na linha de base e 54,1% na segunda onda), que praticavam 150 minutos ou mais por semana de atividades físicas domésticas (55% na linha de base e 70,2% na segunda onda), que praticavam pelo menos 10 minutos e menos de 150 minutos por semana de atividades físicas como deslocamento (40,1% na linha de base e 45,4% na segunda onda) e que não trabalhavam (43,6% na linha de base e 50,7% na segunda onda). Sobre os comportamentos sedentários, a maioria permaneceu com pelo menos três horas diárias de tempo sentado (64,2% na linha de base e 60,4% na segunda onda) e menos de três horas diárias assistindo televisão (67,2% na linha de base e 67,5% na segunda onda). Ao comparar a linha de base com a segunda onda, houve diminuição de indivíduos que permaneceram pelo menos três horas diárias sentados (Tabela 1).

**Tabela 1:** Características sociais, demográficas e comportamentais da amostra estudada (n = 1.241) na linha de base (2014/2015) e na segunda onda (2020/2021). ISA - Atividade Física e Ambiente.

Características	Linha de base (2014/2015)			Segunda onda (2020/2021)			Valor de p *
	n	%	IC95%	n	%	IC95%	
Sociodemográficas							
Sexo							-
Masculino	496	40,0	37,3; 42,7	496	40,0	37,3; 42,7	
Feminino	745	60,0	57,3; 62,7	745	60,0	57,3; 62,7	
Idade (anos)							< 0,001
18-29	209	16,8	14,9; 19,0	130	10,5	8,9; 12,3	
30-39	221	17,8	15,8; 20,0	166	13,4	11,6; 15,4	
40-59	423	34,1	31,5; 36,8	432	34,8	32,2; 37,5	
60 ou mais	388	31,3	28,7; 33,9	513	41,3	38,6; 44,1	
Cor da pele							-
Branca	644	52,3	49,5; 55,1	644	52,3	49,5; 55,1	
Preta	139	11,3	9,6; 13,2	139	11,3	9,6; 13,2	
Amarela	31	2,5	1,8; 3,6	31	2,5	1,8; 3,6	
Parda	371	30,1	27,6; 32,7	371	30,1	27,6; 32,7	
Outras	47	3,8	2,9; 5,0	47	3,8	2,9; 5,0	
Estado marital							0,482
Vive com companheiro(a)	727	58,7	56,0; 61,4	715	57,9	55,2; 60,7	
Vive sem companheiro(a)	511	41,3	38,6; 44,0	519	42,1	39,2; 44,8	
Escolaridade							< 0,001
Até o 5º ano do Ensino Fundamental	308	24,8	22,5; 27,3	253	20,5	18,3; 22,9	
6º ano do Ensino Fundamental a 2ª série do Ensino Médio	296	23,9	21,6; 26,3	267	21,6	19,4; 24,0	
Ensino Médio completo	334	26,9	24,5; 29,5	383	31,0	28,5; 33,7	
Ensino Superior/Pós-graduação incompleto/completo	303	24,4	22,1; 26,9	331	26,8	24,4; 29,4	
Coordenadoria regional de saúde de residência							0,693
Centro	46	3,7	2,8; 4,9	46	3,7	2,8; 4,9	
Leste	225	18,1	16,1; 20,4	227	18,3	16,2; 20,5	
Norte	245	19,7	17,6; 22,1	242	19,5	17,4; 21,8	
Oeste	128	10,3	8,7; 12,1	127	10,2	8,7; 12,1	
Sudeste	295	23,8	21,5; 26,2	291	23,5	21,2; 25,9	
Sul	302	24,3	22,0; 26,8	308	24,8	22,5; 27,3	
Comportamentais							
Práticas de atividades físicas							
No lazer (minutos/semana)							< 0,001
< 10	847	68,3	65,6; 70,8	671	54,1	51,3; 56,8	
≥ 10 e < 150	141	11,4	9,7; 13,3	221	17,8	15,8; 20,0	
≥ 150	253	20,4	18,2; 22,7	349	28,1	25,7; 30,7	
No trabalho (minutos/semana)							0,233
Não trabalha	541	43,6	40,9; 46,4	618	50,7	47,9; 53,5	
< 10	286	23,1	20,8; 25,5	186	15,3	13,3; 17,4	
≥ 10 e < 150	70	5,7	4,5; 7,1	59	4,8	3,8; 6,2	
≥ 150	343	27,7	25,2; 30,2	356	29,2	26,7; 31,8	
Doméstica (minutos/semana)							< 0,001
< 10	330	26,6	24,2; 29,1	157	12,7	11,0; 14,7	
≥ 10 e < 150	229	18,5	16,4; 20,7	211	17,1	15,1; 19,3	
≥ 150	682	55,0	52,2; 57,7	866	70,2	67,6; 72,7	
No deslocamento (minutos/semana)							< 0,001
< 10	486	39,2	36,5; 41,9	383	30,9	28,4; 33,5	
≥ 10 e < 150	498	40,1	37,4; 42,9	563	45,4	42,6; 48,2	
≥ 150	257	20,7	18,5; 23,1	295	23,8	21,5; 26,2	
Comportamentos sedentários							
Tempo sentado ** (horas/dia)							0,019
< 3	442	35,8	33,2; 38,5	424	39,6	36,7; 42,6	
≥ 3	792	64,2	61,5; 66,8	647	60,4	57,4; 63,3	
Tempo assistindo televisão ** (horas/dia)							0,713
< 3	829	67,2	64,5; 69,8	784	67,5	64,7; 70,1	
≥ 3	405	32,8	30,3; 35,5	378	32,5	29,9; 35,3	

IC95%: intervalo de 95% de confiança.

* Teste de Wilcoxon pareado (variáveis categóricas) e teste de McNemar (variáveis dicotômicas);

** Ponderado para representar a semana.

**Figura 2:**
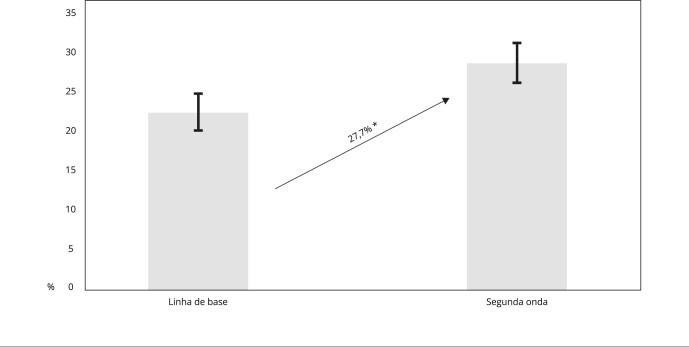
Prevalência de obesidade e seu aumento entre linha de base e segunda onda na amostra estudada (n = 1.241), com indicação de intervalo de 95% de confiança segundo cada período do estudo. ISA - Atividade Física e Ambiente.

A prevalência de obesidade teve um aumento significativo de 27,7% (Figura 2). Nesse período, também foi observado aumento significativo da prevalência de obesidade entre as mulheres, indivíduos com 18 a 29 anos, indivíduos que vivem sem companheiro(a), indivíduos com Ensino Médio completo e indivíduos que praticavam menos de 10 minutos semanais de atividade física no lazer (Tabela 2).

* Diferença significativa entre os períodos.

**Tabela 2:** Análises bivariadas da obesidade com variáveis demográficas, sociais e comportamentais da amostra estudada (n = 1.241) na linha de base (2014/2015) e na segunda onda (2020/2021). ISA - Atividade Física e Ambiente.

Características	Linha de base (2014/2015)			Segunda onda (2020/2021)		
	%	IC95%	Valor de p *	%	IC95%	Valor de p *
Sociodemográficas						
Sexo			0,696			0,124
Masculino	22,04	18,58; 25,94		26,48	22,75; 30,57	
Feminino	22,99	20,09; 26,18		30,56	27,29; 34,03	
Idade (anos)			< 0,001			0,663
18-29	11,71	7,96; 16,89		28,57	21,34; 37,10	
30-39	18,72	14,08; 24,46		26,88	20,56; 34,30	
40-59	29,16	24,97; 33,72		31,06	26,83; 35,63	
60 ou mais	23,58	19,60; 28,08		27,80	24,04; 31,90	
Cor da pele			0,097			0,020
Branca	22,03	18,98; 25,42		26,62	23,31; 30,22	
Preta	30,15	23,00; 38,41		36,03	28,38; 44,46	
Amarela	10,00	3,19; 27,23		19,35	8,84; 37,27	
Parda	21,86	17,91; 26,40		29,25	24,76; 34,18	
Outras	27,27	16,08; 42,33		44,44	30,61; 59,20	
Estado marital			0,001			0,031
Vive com companheiro(a)	26,01	22,93; 29,35		31,25	27,93; 34,78	
Vive sem companheiro(a)	17,89	14,78; 21,50		25,55	21,91; 29,56	
Escolaridade			0,107			0,313
Até o 5º ano do Ensino Fundamental	26,91	22,19; 32,22		32,78	27,13; 38,98	
6º ano do Ensino Fundamental a 2ª série do Ensino Médio	22,11	17,72; 27,23		26,25	21,24; 31,97	
Ensino Médio completo	18,73	14,88; 23,31		30,16	25,73; 34,99	
Ensino Superior/Pós-graduação incompleto/completo	23,08	18,64; 28,21		26,91	22,37; 31,99	
Coordenadoria regional de saúde de residência			0,445			0,702
Centro	26,09	15,35; 40,72		30,43	18,81; 45,24	
Leste	20,18	15,36; 26,06		27,73	22,20; 34,03	
Norte	24,08	19,12; 29,85		28,81	23,38; 34,94	
Oeste	16,54	11,01; 24,08		24,00	17,29; 32,30	
Sudeste	23,29	18,78; 28,50		28,62	23,64; 34,18	
Sul	24,58	20,00; 29,81		31,89	26,86; 37,39	
Comportamentais						
Práticas de atividades físicas						
No lazer (minutos/semana)			0,012			< 0,001
< 10	24,16	21,38; 27,19		33,69	30,15; 37,43	
≥ 10 e < 150	25,71	19,13; 33,62		25,81	20,41; 32,06	
≥ 150	15,66	11,65; 20,74		21,90	17,85; 26,57	
No trabalho (minutos/semana)			0,556			0,968
Não trabalha	21,80	18,49; 25,52		28,57	25,08; 32,34	
< 10	25,70	20,94; 31,12		30,43	24,20; 37,48	
≥ 10 e < 150	20,29	12,35; 31,50		29,82	19,33; 42,98	
≥ 150	21,83	17,74; 26,55		28,90	24,39; 33,85	
Doméstica (minutos/semana)			0,281			0,494
< 10	25,46	21,01; 30,48		33,11	26,05; 41,03	
≥ 10 e < 150	19,91	15,20; 25,65		29,06	23,21; 35,70	
≥ 150	22,14	19,16; 25,44		28,35	25,42; 31,48	
No deslocamento (minutos/semana)			0,027			0,033
< 10	26,61	22,85; 30,75		34,06	29,38; 39,07	
≥ 10 e < 150	19,88	16,57; 23,66		26,50	22,97; 30,35	
≥ 150	20,31	15,81; 25,70		26,96	22,18; 32,35	
Comportamentos sedentários						
Tempo sentado ** (horas/dia)			0,056			0,059
< 3	19,68	16,21; 23,68		25,54	21,57; 29,97	
≥ 3	24,46	21,56; 27,60		30,93	27,45; 34,63	
Tempo assistindo televisão ** (horas/dia)			0,010			0,005
< 3	20,27	17,65; 23,17		26,14	23,15; 29,38	
≥ 3	26,82	22,69; 31,39		34,14	29,49; 39,12	

IC95%: intervalo de 95% de confiança.

* Teste de qui-quadrado;

** Ponderado para representar a semana.

Quando os fatores associados à obesidade foram analisados longitudinalmente, foi observado que indivíduos que praticaram pelo menos 150 minutos semanais de atividade física no lazer e os que praticaram pelo menos 10 minutos e menos de 150 minutos semanais de atividade física como deslocamento tiveram 56% e 51% de proteção contra a obesidade, respectivamente. Assim como os indivíduos que vivem sem companheiro(a) tiveram 53% de proteção quando comparados aos indivíduos com companheiro(a). Indivíduos com idade entre 40 e 59 anos, apresentaram cinco vezes mais chance de terem obesidade quando comparados aos com 18 a 29 anos; bem como indivíduos com cor de pele preta, que apresentaram 4,7 vezes mais chance de ter obesidade quando comparados aos de cor de pele branca. O comportamento de tempo assistindo televisão ficou próximo do limite de significância de associação longitudinal com a obesidade (p = 0,059) (Tabela 3).

**Tabela 3:** Modelo multinível de regressão logística para análise longitudinal dos fatores sociodemográficos e comportamentais relacionados com a obesidade em amostra de adultos paulistanos (n = 1.241). ISA - Atividade Física e Ambiente.

Variáveis	Modelo multinível *		
	OR	IC95%	Valor de p
Atividades físicas no lazer (minutos/semana)			
< 10	Referência	Referência	Referência
≥ 10 e < 150	0,91	0,50; 1,66	0,758
≥ 150	0,44	0,26; 0,76	0,003
Atividades físicas de deslocamento (minutos/semana)			
< 10	Referência	Referência	Referência
≥ 10 e < 150	0,49	0,30; 0,80	0,004
≥ 150	0,66	0,38; 1,17	0,156
Tempo de televisão ** (horas/dia)			
< 3	Referência	Referência	Referência
≥ 3	1,57	0,98; 2,51	0,060
Tempo sentado ** (horas/dia)			
< 3	Referência	Referência	Referência
≥ 3	1,39	0,86; 2,26	0,180
Idade (anos)			
18-29	Referência	Referência	Referência
30-39	1,75	0,72; 4,23	0,216
40-59	5,00	2,02; 12,38	< 0,001
60 ou mais	2,04	0,82; 5,10	0,126
Sexo			
Masculino	Referência	Referência	Referência
Feminino	1,43	0,82; 2,51	0,208
Cor da pele			
Branca	Referência	Referência	Referência
Preta	4,70	1,85; 11,95	0,001
Amarela	0,27	0,04; 1,75	0,169
Parda	1,30	0,69; 2,44	0,412
Outras	3,86	0,89; 16,86	0,072
Estado marital			
Vive com companheiro(a)	Referência	Referência	Referência
Vive sem companheiro(a)	0,47	0,28; 0,78	0,004
Escolaridade			
Até 5º ano do Ensino Fundamental	Referência	Referência	Referência
6º ano do Ensino Fundamental a 2ª série do Ensino Médio	0,55	0,25; 1,17	0,120
Ensino Médio completo	0,68	0,32; 1,48	0,335
Ensino Superior/Pós-graduação completo/incompleto	0,96	0,42; 2,19	0,928

IC95%: intervalo de 95% de confiança; OR: *odds ratio*.

Nota: valores em negrito = teste Wald, p < 0,05.

* Modelo multinível com dois níveis: observações (n = 2.428) e indivíduos (n = 1.241) e ajustado para variável tempo; ** Ponderado para representar a semana.

## Discussão

Os principais resultados deste estudo mostraram que praticantes de atividade física no lazer e como forma de deslocamento, bem como pessoas que vivem sem companheiro(a) diminuíram as chances de ter obesidade. Por outro lado, pessoas de cor da pele preta e com 40 a 59 anos apresentaram maior chance de ter obesidade.

O aumento da prevalência de obesidade de 27,7%, observado na amostra no período de 5 a 7 anos, foi maior do que o aumento de 18,1% em estudo de coorte com adultos noruegueses em um período de oito anos ^24^, e do que o aumento de 23,8% observado em estudo longitudinal com adultos australianos em um período de 14 anos ^25^. Estudo brasileiro com dados de coortes de nascimentos realizados em diferentes regiões do Brasil também demonstrou aumento nas prevalências de obesidade em adultos. Em Ribeirão Preto, São Paulo, foi de 12,2% para 35%, entre 2002 e 2017 na coorte de nascimento de 1978/1979; e em Pelotas, Rio Grande do Sul, foi de 4,8% para 22,9%, entre 2000 e 2012 na coorte de nascimento de 1982, e de 9,1% para 16,9%, entre 2011 e 2015 na coorte de nascimento de 1993 ^26^. Provavelmente, o aumento observado neste estudo foi menor por abranger diferentes faixas etárias da vida adulta, já que não se trata de uma coorte de nascimento, e ocorrer em uma megacidade, repleta de importantes desigualdades sociais. O padrão de aumento da obesidade observado nos escassos estudos longitudinais brasileiros, também ocorre em estudos nacionais de tendência temporal, como o Vigitel ^5^ e a *Pesquisa Nacional de Saúde* ^27^. Além disso, a prevalência de obesidade na segunda onda do estudo está alinhada à prevista para 2020 em adultos e idosos do Município de São Paulo, de acordo com pesquisa epidemiológica com dados do inquérito ISA-Capital de 2003 a 2015 ^28^.

Neste estudo, sexo e escolaridade não foram associados longitudinalmente com a obesidade. Mas foi observado aumento significativo de sua prevalência entre as mulheres e entre os indivíduos com ensino médio completo. Aumento que também foi observado no sexo feminino mostrado nos relatórios de 2014 e 2021 do Vigitel, indo de 16,7% para 24,2%, respectivamente ^29^. É possível que esta amostra esteja em processo de transição da “epidemia de obesidade”, no qual não há diferença entre sexos e as mudanças das prevalências entre os níveis socioeconômicos, incluindo escolaridade, esteja no processo de inverter sua relação ^7^. Outro fator que pode ter influenciado é o aumento geral do nível de escolaridade da população brasileira nos últimos anos, com destaque para as categorias de Ensino Médio completo e Ensino Superior incompleto e completo ^30^, que também foi identificado nesta amostra, o que pode ter mascarado ou suavizado a relação entre nível educacional e obesidade.

Contudo, a cor da pele autorreferida foi identificada como um fator sociodemográfico associado longitudinalmente à obesidade. O Brasil é um país miscigenado com diversas descendências presentes, e, entre elas, ainda ocorre maior vulnerabilidade socioeconômica entre indivíduos de cor de pele preta ^31^. Em estudo longitudinal com adultos brasileiros, foi observado relação entre cor de pele preta, discriminação social e aumento do risco de ganho de peso e aumento do IMC ^32^. Em outro estudo brasileiro, esse transversal, foi identificada a relação entre obesidade e cor da pele; contudo, ela varia a depender do sexo e do nível socioeconômico, destacando uma relação mais forte entre mulheres ^33^. Essa relação entre cor de pele e obesidade também foi observada em adultos estadunidenses, expondo que esses resultados refletem, em parte, as diferenças e vantagens sociais relacionadas à cor da pele ^34^ ^,^ ^35^. Além disso, também deve-se considerar que o aumento da autodeclaração de cor de pele preta observado na população brasileira na última década ^36^, devido a movimentos de conscientização e políticas afirmativas de identidade racial, pode ter influenciado o aumento da prevalência de obesidade nessa população específica e auxiliado a identificar, de forma mais fidedigna, essa relação longitudinal.

No que tange à idade, com o seu avançar há um aumento na prevalência de obesidade, apresentando pico entre a meia-idade e a fase idosa ^6^. Isso corrobora os resultados observados, em que a idade foi um fator associado à obesidade, principalmente para a faixa etária de 40 e 59 anos. Uma hipótese é a de que a partir dos 30 anos há o início da perda de massa muscular, seguido de declínio de certas funções fisiológicas e mudanças hormonais ^37^, em que a meia-idade abraça o início de grande parte dessas alterações, além da transição para a terceira idade. Além disso, ainda que os grupos de indivíduos de 18 a 29 anos não tenham apresentado associação estatisticamente significativa para a obesidade nas análises longitudinais, foi observado que a prevalência de obesidade nessa faixa etária mais que dobrou no período. Esse aumento também foi observado nos dados disponibilizados pelo Vigitel entre 2014 e 2021, no qual, entre a faixa etária de 18 a 29 anos, foi de 46%, mas, analisando as faixas de 18 a 24 anos, foi observado um aumento de 2,4 vezes na prevalência de obesidade nesse mesmo período ^29^. Logo, destaca-se a necessidade de estudos que investiguem o comportamento da obesidade em adultos jovens.

Além da mudança etária ao longo do tempo, deve-se considerar as mudanças no contexto social, como as esferas de família e trabalho. Por exemplo, o estado marital foi associado longitudinalmente à obesidade, sendo que o fato de viver com companheiro(a) aumentou as chances de ter obesidade. Algumas evidências longitudinais sugerem que começar a viver com o(a) companheiro(a) aumenta as chances de desenvolver obesidade ^38^ ^,^ ^39^. Cobb et al. ^38^ observaram, em estudo de coorte, que adultos casados sem obesidade apresentaram maior risco de desenvolver obesidade se o parceiro também desenvolvesse obesidade. Essa relação entre obesidade e estado marital pode ser reflexo do compartilhamento de rotina e influência de comportamentos. Por exemplo, viver com o(a) companheiro(a) proporciona mais oportunidades para ingestão alimentar, pois casais tendem a se alimentar na companhia um do outro, o que pode contribuir para o aumento da ingestão energética ^40^. Outra característica observada na literatura é que começar a viver com o(a) companheiro(a) foi associado à redução da prática de atividade física ^39^. Além disso, é possível que indivíduos sem companheiro(a) podem estar mais preocupados no mantimento do estado nutricional e o reflexo na sua aparência física ^39^ ^,^ ^40^, do que indivíduos em um relacionamento.

A respeito dos fatores comportamentais, como observado na literatura majoritariamente proveniente de países de alta renda, as pessoas mais ativas fisicamente no lazer e deslocamento tiveram proteção contra a obesidade ^10^. É importante destacar que há uma escassez de estudos longitudinais, principalmente os que incluem análises dos quatro domínios de atividade física separadamente em países de média e baixa renda ^10^. Alinhados às recomendações internacionais ^41^, com outros estudos longitudinais realizados na Austrália e no Reino Unido ^42^ ^,^ ^43^, e à revisão sistemática e meta-análise realizada por Wu et al. ^44^, os resultados desta pesquisa mostram a importância da promoção da atividade física nos domínios de lazer, como as práticas de exercícios físicos e esportes, e de deslocamento, como as caminhadas e uso de bicicleta, em adultos que vivem em país de renda média em economias em fase de transição; pois, além da diminuição da obesidade, também podem contribuir com a melhora da saúde nas cidades ^41^ ^,^ ^44^.

Os comportamentos sedentários não foram associados longitudinalmente à obesidade, destacando que o tempo assistindo televisão ficou no limite da significância dessa relação com fator para aumentar as chances. Estudos prospectivos conduzidos nos Estados Unidos ^45^ e no Reino Unido ^46^ encontraram associações significativas entre a obesidade e o tempo assistindo televisão; porém, esse tipo de comportamento é complexo e se relaciona com outras características como o contexto alimentar e a exposição a programas e propagandas, influenciando na qualidade e na quantidade da ingestão alimentar, bem como em mecanismos de saciedade ^46^. Essa complexidade é observada na literatura, na qual ainda não há convergência dessa relação longitudinal ^47^.

Vale destacar que a segunda onda do estudo abrangeu a época de pandemia de COVID-19, na qual foram impostas restrições para evitar a proliferação da doença, como o distanciamento social, que culminaram em alterações nos comportamentos ^48^ e no reporte de peso corporal ^49^ da população. O Brasil foi um dos países mais acometidos pela pandemia de COVID-19 ^50^. Isso reflete na cidade de São Paulo, a mais populosa do país, que teve o maior número de óbitos ^51^. Portanto, os resultados observados podem ter sofrido influência da pandemia, colaborando para o aumento da prevalência de obesidade observado no período.

Este estudo apresenta limitações: (i) a realização de entrevistas telefônicas na segunda onda devido à pandemia de COVID-19, culminando em 35,5% da amostra original entrevistada e diferenças significativas na escolaridade, sexo, idade e no desfecho de obesidade que podem ter prejudicado as análises de alguns fatores e sua relação longitudinal com obesidade; (ii) houve somente dois períodos de avaliação, com distância de 5 a 7 anos entre eles, podendo prejudicar análises mais detalhadas envolvendo as nuances dos comportamentos avaliados; e (iii) não houve avaliação do consumo alimentar, que está intrinsecamente relacionado ao balanço energético, tanto quantitativamente quanto qualitativamente, pois entende-se que a dieta está relacionada a desfechos nutricionais, inclusive a obesidade, podendo ter causado uma superestimação do efeito da atividade física como fator protetor de obesidade.

Apesar das limitações, este estudo é um dos poucos com delineamento longitudinal que foi conduzido com adultos vivendo em país de renda média-alta e em uma megalópole como São Paulo, investigando de forma detalhada as relações da obesidade com os quatro domínios da atividade física. Outro ponto forte foram as análises de dados que levaram em conta a característica longitudinal das observações ao utilizar modelos multiníveis longitudinais.

## Conclusão

Foi identificado aumento de 27,7% de obesidade na amostra de adultos paulistanos durante o intervalo de 5 a 7 anos de estudo. A faixa etária de 40 a 59 anos e a cor de pele preta foram associados com maiores chances de obesidade, enquanto o estado marital caracterizado com viver sem companheiro(a), praticar atividades físicas de deslocamento e pelo menos 150 minutos semanais de atividade física no lazer diminuíram as chances de obesidade. Os resultados contribuem para o melhor entendimento desse problema de saúde pública e podem respaldar ações, programas e políticas públicas de controle da obesidade, principalmente intervenções para a promoção da atividade física no tempo de lazer e como forma de deslocamento.
